# Neuroinflammation-on-a-chip for multiple sclerosis research: a narrative review

**DOI:** 10.1097/MS9.0000000000002231

**Published:** 2024-05-28

**Authors:** Christin Berjaoui, Charbel Kachouh, Safaa Joumaa, Mohammad Hussein Ghayyad, Bisrat Abate Bekele, Rita Ajirenike, Zeina Al Maaz, Sara Awde, Magda Wojtara, Abubakar Nazir, Olivier Uwishema

**Affiliations:** a Oli Health Magazine Organization, Research, and Education, Kigali, Rwanda; b Faculty of Medicine, Beirut Arab University; c Faculty of Medicine, Saint-Joseph University; d Faculty of Medical Science, Lebanese University, Beirut, Lebanon; e College of Health Sciences, Addis Ababa University, Addis Ababa, Ethiopia; f Department of Internal Medicine, Rivers State University Teaching Hospital, Rivers State, Nigeria; g University of Michigan Medical School, Ann Arbor, MI; h Clinton Global Initiative University, New York, NY, USA; i Department of Medicine, King Edward Medical University, Lahore, Pakistan; j Faculty of Medicine, Karadeniz Technical University, Trabzon, Turkey

**Keywords:** chip, multiple sclerosis, neuroinflammation

## Abstract

**Introduction:**

Multiple sclerosis (MS) is a chronic inflammatory condition that impacts the central nervous system. It is distinguished by processes like demyelination, gliosis, neuro-axonal harm, and inflammation. The prevailing theory suggests that MS originates from an immune response directed against the body’s own antigens within the central nervous system.

**Aim:**

The main aim of this research paper “Neuroinflammation-on-a-Chip” for studying multiple sclerosis is to enhance our comprehension of MS development, demonstrate the application of cutting-edge technology, and potentially provide valuable insights for therapeutic approaches.

**Methods:**

The available literature for this Narrative Review was searched on various bibliographic databases, PubMed, NCBI, and many other medical references using an individually verified, prespecified approach. Studies regarding the significance of MS and its neuroinflammatory pathogenesis in addition to the development and optimization of neuroinflammatory-on-a-chip and the advancement in innovations in this field have been reviewed in this research for a better understanding of “Neuroinflammation-on-a-chip for multiple sclerosis”. The level of evidence of the included studies was considered as per the Centre for Evidence-Based Medicine recommendations.

**Results:**

Several studies have indicated that the brain-chip model closely mimics cortical brain tissue compared to commonly used conventional cell culture methods like the Transwell culture system. Additionally, these studies have clearly demonstrated that further research using brain chips has the potential to enhance our understanding of the molecular mechanisms and roles of blood-brain barrier (BBB) transporters in both normal and disease conditions.

**Conclusion:**

Understanding neuroinflammation processes remains essential to establish new MS treatments approaches. The utilization of brain chips promises to advance our understanding of the molecular processes involving BBB transporters, both in normal and diseased states. Further research needs to be addressed in order to enhance the performance and understanding of neuroinflammation on a chip, hence aiming to provide more effective treatments for all CNS diseases.

## Introduction

Highlights

- Multiple sclerosis (MS) is characterized by the immune system mistakenly attacking the central nervous system, leading to inflammation, demyelination, and a range of symptoms.
- To address the challenges posed by MS, researchers require advanced tools and technologies to uncover its underlying mechanisms and develop novel therapeutic strategies such as advanced genetic and transcriptomic analysis and biomarker discovery.
- A novel microfluidic platform has been created for the purpose of conducting preliminary research into the infiltration of monocytes into the central nervous system (CNS). This innovative system holds great potential as a robust tool for the analysis and screening of brain inflammation, facilitating the exploration of multiple neurodegenerative diseases.
- The utilization of brain chips promises to advance our understanding of the molecular processes involving blood-brain barrier (BBB) transporters, both in normal and diseased states.

Multiple sclerosis (MS) is an autoimmune persistent condition where the immune system incorrectly targets and damages the protective myelin sheath around the nerve fibres in the central nervous system (CNS). This demyelination process interrupts the regular transmission of electrical signals through the nerves. This disorder is influenced by genetic, environmental, and immunological elements. MS ranks among the most prevalent neurological disorders afflicting individuals in their early adulthood. Its occurrence differs by location, with greater frequencies found in temperate zones, like North America and Europe^1–3^. MS can present itself in diverse ways, giving rise to a broad spectrum of symptoms. These can encompass sensations of tiredness, muscle weakness, challenges with mobility, sensations of numbness or tingling, issues with vision, and difficulties in cognitive function, which can affect individual quality of life in which lies the importance of detecting disease early and treating it^4,5^. MS is a chronic inflammatory disorder affecting the central nervous system, characterized by processes such as demyelination, gliosis, neuro-axonal damage, and inflammation. It is currently believed to result from an autoimmune response against self-antigens within the central nervous system, particularly in individuals with a genetic predisposition, with autoreactive T cells, B cells and myeloid cells working together in the immunopathological processes of the disease^6^. Innovative research tools are critical in advancing our understanding of MS and developing more effective treatments for this complex neurological disease. MS is characterized by the immune system mistakenly attacking the central nervous system, leading to inflammation, demyelination, and a range of symptoms. To address the challenges posed by MS, researchers require advanced tools and technologies to uncover its underlying mechanisms and develop novel therapeutic strategies as advanced imaging technique, genetic and transcriptomic analysis also biomarker discovery^7–9^. The neuroinflammation on a brain-chip model for multiple sclerosis is made to study the pathogenesis of neurodegenerative diseases as well as discovering new and the most effective treatments through clinical research, that will help to reach the most effective medical treatment and to perceive the progression of the disease even better and that all happens through the way the brain-chip works that is similar to the adult human cortex as it is rich in important brain pathways. When the chip is exposed to TNF-α, it will make a similar inflammation as that happens in that brain then it will secrete inflammatory molecules after activating glial cells, thus affecting barrier function^10^. The main aim of this research paper “Neuroinflammation-on-a-Chip” for studying Multiple sclerosis is to enhance our comprehension of MS development, demonstrate the application of cutting-edge technology, and potentially provide valuable insights for therapeutic approaches.

## Understanding multiple sclerosis and neuroinflammation

**Figure 1:**
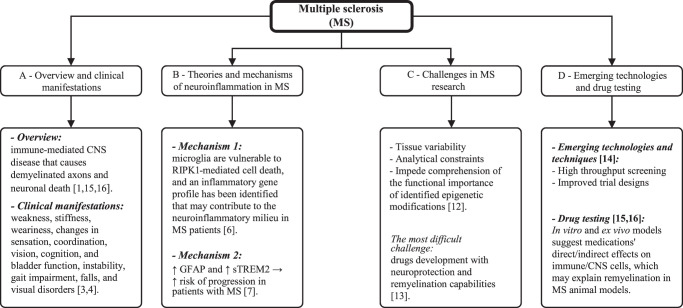
Figure provides an overview of multiple sclerosis (MS), including its clinical. manifestations (A), theories and mechanisms of MS neuroinflammation (B), challenges in MS research (C), and emerging technologies and drug testing related to MS (D). CNS, central nervous system; GFAP, glial fibrillary acid protein; RIPK1, receptor-interacting protein kinase 1; sTREM2, soluble triggering receptors expressed on myeloid cells-2.

MS is an immune-mediated CNS disease that causes demyelination and axonal/neuronal death, resulting in characteristic multifocal lesions in both grey and white matter visible on MRI^11^. Categorization of MS phenotypes can be complex, thus new adjustments in three clinical stages have been proposed to clarify present classifications: a pre-clinical stage that can only be diagnosed by MRI, a relapsing-remitting stage (RRMS) marked by periods of neurologic dysfunction followed by remission, and a progressive stage that typically develops from the relapsing stage^12–14^. MS is diagnosed using McDonald’s diagnostic criteria, which relate clinical presentation to distinctive lesions shown by MRI, cerebrospinal fluid (CSF) studies, and visual evoked potentials^12^. Lesions grow in various sections of the CNS, causing symptoms such as weakness, stiffness, and weariness, as well as changes in sensation, coordination, vision, cognition, and bladder function. As a result, people with multiple sclerosis are more prone to develop instability, gait impairment, and falls^15^. MS damages the nervous system’s myelinated parts, such as the optic nerves, cerebellum, brainstem, and spinal cord, resulting in visual difficulties^16^. Despite advances in immunology, cell biology, and genetics over the last decades, the main cause of MS and its triggering components remains unclear^17^. However, receptor-interacting protein kinase 1 (RIPK1) may be cause a neuroinflammation mechanism and may be related to MS pathogenesis since it mediates cell death and inflammatory signalling and was found to be increased during recorded results^18^. Another study advocates that neuroinflammation may be associated with glial fibrillary acid protein (GFAP) and soluble triggering receptors expressed on myeloid cells-2 (sTREM2) levels in multiple sclerosis, with the findings indicating that increased GFAP and sTREM2 expression may characterise patients at higher risk of progression, as they were positively related to age at diagnosis^19^. Advances in MRI and serological and genetic testing have improved the accuracy of identifying multiple sclerosis from these illnesses, yet misdiagnosis can still occur^20,21^. There is methodological limits since there is a variances between individuals and tissues, and their analysis. As a result it is hard to comprehend the significance of the identified changes in gene activity that leads to the difficulty in explaining the biology behind them^22^. The remaining challenge for multiple sclerosis is the development of medicines that mix neuroprotection and remyelination to treat and, eventually, prevent progressive forms of the disease^23^. The most notable advances in the MS field have been modern technologies for discovering components, such as high throughout screening, and improved trial designs and outcomes to assess potential neuroprotective advantages better^24^. Given that MS causes continuously demyelinated axons, finding medicines that induce remyelination of deprived axons will be critical. In vitro and ex vivo models show that these medications have direct or indirect effects on the immune system and CNS cells, which may help explain how they cause remyelination in animal models of MS^25,26^ (Fig. 1).

## Neuroinflammation-on-a-chip technology

Microfluidic chip technology, commonly known as “labs on a chip” or micro total analysis systems have advanced quickly in the last several years. In addition to facilitating the simultaneous operation and analysis of cells cultivated on the fully integrated and automated chips from a single cell level to a large cell population, microfluidic cell culture may manage micro- and nano-liter fluid flow in precisely defined geometry^27^.

Microfluidics-based methods for developing perfusable vascular networks can be broadly divided into two categories: (a) self-assembly (inducing a microvessel network to self-organize through either angiogenesis or vasculogenesis and achieving a stable anastomosis with adjacent microfluidic channels) and (b) vascular patterning^28^.

Different cell types join two or more microchannels through porous membranes to replicate the interface between the various tissues that makes up the blood-brain barrier. Physical forces, such as mechanical compression, periodic strain, and shear stress at physiologically appropriate levels, can be incorporated into these systems. Additionally, they examine how medications or poisons cause organ-specific reactions, such as the mobilization of circulating immune cells. It has been discovered that controlling the fluid flow inside these chips is quite helpful. When the microfluidic channel diameter is less than ~1 mm, the flow is laminar because viscous forces predominate over inertial forces on a tiny length scale. It was possible to analyze transcytosis, absorption, and tissue barrier function by integrating porous substrates to divide the two microchannels^29^. The goal of microfluidic devices is to develop more *in vivo*-like systems while avoiding modifications to cell functions that differ from those seen in nature. This can be achieved more easily in microfluidic devices than in conventional instruments by regulating the microenvironment (such as the cell matrix, flow rate, chemical gradients, pH, temperature, etc.). Cell-based tests using microfluidic technologies may improve the biological relevance of cell models without sacrificing or requiring more throughput than existing techniques. Microfluidic-based cell cultures, in contrast to traditional cell culture techniques, offer a number of appealing advantages, including a continuous supply of nutrients, waste removal, schedule flexibility, liquid handling systems, and a high degree of automation. This can be achieved more easily in microfluidic devices than in conventional instruments by regulating the microenvironment (such as the cell matrix, flow rate, chemical gradients, pH, temperature, etc.). The development of microfluidics brought significant innovation to the manipulation and preservation of biosamples in cell culture^30^. The Blood-Brain Barrier-on-a-Chip with Neuroinflammation and the Micro-engineered Human Brain-Chip Platform of the Neurovascular Unit are two instances of current neuroinflammation^10,29^.

## Applications in MS research

Brain-on-a-chip (BoC) integrates a chemotaxis module and provides a controlled means of elucidating the interactions between neurons and glia, as well as recapitulating the pathological signatures of commonly occurring neurological disorders. Furthermore, BoC enables three-dimensional selective neuro-glial engagement and can distinguish disease-associated microglia from a heterogeneous population. Benefits of understanding the relationships between neurons and glia are revealed, along with the risk factor for synaptic dysfunction and neuronal loss that is the significant activation of innate immune cells. When all taken together, BoCs provide trustworthy and useful frameworks for examining pathogenic pathways and evaluating treatment approaches for neurological diseases^31^.

MS is a chronic neurodegenerative disease characterized by axonal myelin loss in the CNS. an autoimmune inflammatory condition characterized by the recruitment of self-reactive lymphocytes, primarily CD4+ T cells, cytokines, and chemokines, in CNS, contributing to the collapse of the blood-brain barrier (BBB),and activation of local astrocytes and microglia. Several varieties of Th cells are linked, such as Th1, Th17, Th1-like Th17, Th9, and Th22^32^. This concept has been supported by the experimental autoimmune encephalomyelitis (EAE)^33^.

Observations in MS patients show a microglial dysfunction caused by overactivity of the mitogen-activated protein kinase (MAPK^ERK^) pathway. This disrupts local oligodendrocytes, leading to locoregional demyelination, a characteristic of MS^34^.

A lot of drugs have been screened, Natalizumab is a monoclonal antibody that targets α4 integrin, lowers T-cell trafficking to the CNS, and reduces relapse rate^35^. In addition, it was confirmed an advantageous impact of B-cell depletion therapy^33^.

A case report included Three paediatric RRMS patients who were either not responding well to previous immunomodulatory treatments or whose side effects were serious. Received Every 4 weeks, natalizumab reports no more relapses and a notable improvement in their quality of life. No new T2-weighted lesions or gadolinium-enhancing lesions were detected on follow-up MR imaging^36^.

Fingolimod is a prodrug metabolized to a sphingosine-1-phosphate (S1P) analogue in the body. It decreases S1P receptor 1 on leucocytes and endothelial cells, trapping naive and memory T lymphocytes. Lowering disease activity by reducing relapse rates and MRI-visible activity^35^. Teriflunomide and dimethyl-fumarate are oral immunomodulatory medications that have been approved as well as alemtuzumab, a leucocyte-depleting CD52 antibody. Other drugs in advanced clinical trials include laquinimod and daclizumab (anti-CD25 antibodies), ocrelizumab, and ofatumumab (both CD20 antibodies targeting B lymphocytes)^35^.

Several possible biomarkers have been postulated, but their clinical value remains uncertain, and none is currently acknowledged as sensitive and reliable^37^. However, investigations show that sNfL levels are increased in MS patients, and they correlate with the presence and activity of localized lesions in the brain and spinal cord^37^.

According to a case report, an MS patient experienced an exacerbation during week 15 of the research; nevertheless, when the NfL levels were later investigated, they revealed a nearly three-fold increase in CSF NfL levels prior to symptoms at week 6^38^.

Moreover, the presence of Gadolinium-enhancing lesions on MRI in MS is indicative of ongoing inflammation, and lesion burden and is prognostic for relapse initiation and severity. The link between grey matter atrophy and cognitive dysfunction, suggests a biomarker for clinical severity^35^. Besides that, several antigens, including myelin oligodendrocyte glycoprotein (MOG), proteolipid protein (PLP), myelin basic protein (MBP), myelin-associated glycoprotein (MAG), myelin-associated oligodendrocytic basic protein (MOBP), have been studied as potential targets of T-cell and B-cell responses in MS. However, those T lymphocytes have been also seen in healthy people^33^.

Oligoclonal bands (OCB), immunoglobulin bands, are formed by plasma cells in CNS producing immunoglobulin G (IgG) and M (IgM). Its presence in CSF but not in serum is a significant signal of intrathecal antibody production and is detected in nearly all MS patients, but not limited to MS^39^.

**Figure 2:**
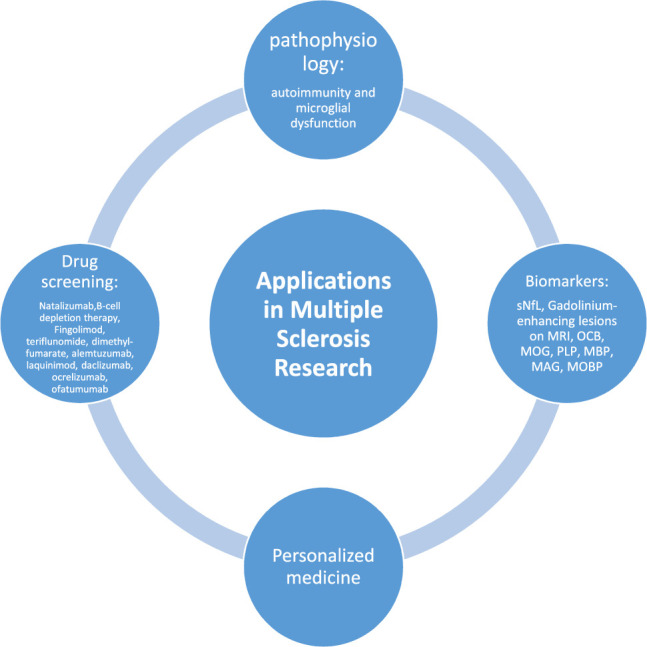
Figure represents the summary of applications in multiple sclerosis research. MAG, myelin-associated glycoprotein; MBP, myelin basic protein; MOBP, myelin-associated oligodendrocytic basic protein; MOG, myelin oligodendrocyte glycoprotein; OCB, oligoclonal bands; PLP, proteolipid protein; sNFL, serum neurofilament light chain.

Given the variety of clinical features, personalized treatment is desirable. The determination of alter therapies relies on evidence-based prognostication, initial treatment choice, and evaluation of early treatment responses. Baseline clinical, environmental, and demographic factors, MRI measures, and biomarkers all influence prognosis. In addition to patient-related issues such as comorbidities, pregnancy planning, patient preferences, and risk tolerance, as well, as drug-related factors^40^ (Fig. 2).

## Development and optimization of a neuroinflammation-on-a-Chip

The iPSC-derived brain cells as well as complex multi-layered three-dimensional organoids, which are considered a marked progression in the field of stem cell biology, have helped in establishing the latest human cell templates necessary for investigation. The combination of iPSC-derived differentiated cells and the organ-on-chip hardware led to the establishment of a human brain-chip template that aids in detecting as well as tracking inflammation. Two microfluidic channels, referred to as the top and bottom channels, are separated by a thin porous polydimethylsiloxane (PDMS) membrane that constitutes the brain-chip. The PDMS works by reinforcing the extracellular matrix (ECM) and aiding in cellular communication. The brain channel of the chip, also known as the top channel, is responsible for harbouring the co-culture of essential materials of the neurovascular unit (NVU), and these include microglial cells, astrocytes, pericytes, and excitatory and inhibitory cortical neurons. As for the vascular channel, or the bottom channel, it is planted with human iPSC-derived brain microvascular endothelial cells (iBMECs), that shape a lumen-like anatomy that resembles the interconnection between the brain parenchyma and circulation^10^.

Neuroinflammation occurs in numerous neurodegenerative diseases, and although the pathophysiology of neuroinflammation against pathogens was established, the particular cause for glial cells and astrocyte generation is not quite understood. As a result, in order to demonstrate neuroinflammation in the brain, and to confirm the superiority of the existence of astrocytes and microglial cells in the channels, a study was conducted where TNF-alpha was immediately infused into the brain channel at a constant concentration for two days. It showed that the concentrations of interleukin-1beta, interleukin-6, and interferon-gamma were increased, confirming the microglia-astrocyte-dependent inflammatory responses to TNF-alpha in the brain-chip. It moreover demonstrates the brain-chip’s capability to identify particular changes in the cell-cell communications that comprise the expansion and advancement of neuroinflammation^10^. These results demonstrate the application of the brain-chip to detect and specify changes in cell-cell interactions in the presence of various disorders^10^.

Embryonic stem cell transplantations in the clinical practice are being replaced by iPSC due to their significant efforts in understanding diseases and screening for potential medications intoxication. In fact, one of its advantages is the ability to implement it without the limitations of donor cells’ access upon investigation different diseases^41^.

## Case studies on neuroinflammation-on-a-chip for MS research

Owing to the novelty of neuroinflammation-on-a-chip technology, the number of published studies is less than a handful. One study done in 2016 by Herland A. and colleagues illustrated the clear-cut input of astrocytes and pericytes in the pathophysiology of neuroinflammation. This was identified in a 3D human BBB on a chip. This study clearly demonstrated the superiority of the microfluidic BBB chip in ensuring a sustained and increased release of G- CSF and IL-6 to far higher levels when compared to the amount seen while using the static Transwell brain model which happens to be the most frequently used cell culture system for modelling brain in vitro^42^. The use of BBB chip illustrated that astrocytes and pericytes can on their own cause elevations in G- CSF and IL-6 when cultured with endothelium regardless of basal unstimulated conditions. In this study, it was highlighted that having only pericytes was enough to initiate an increase in the baseline G-CSF levels in a 3D- BBB chip model^42^.

In another study by Pediaditakis.and colleagues in 2022, a micro-engineered brain-chip to model neuroinflammation in humans. It was noted that the adult human cortex manifested a lower transcriptomic similarity to the brain chips when compared to the Transwell models^10^. This discovery thus demonstrates that the brain-chip epitomizes the cortical brain tissue more closely than the frequently used and conventional cell culture system like the Transwell culture. This study has clearly shown that more research in brain-chip is bound to increase the comprehension of the molecular basis and function of the BBB transporters within basal and disease states^10^.

These case studies show the possibility of developing new therapeutic interventions that target these responses as illustrated in the BBB chip. Also, there is a clear demonstration of the potential for the incorporation of microglia and astrocytes in novel pharmaceutical discoveries for the management of multiple sclerosis^10,42^. These are the propelling reasons for this narrative review

## Challenges and future directions

### Technical challenges and limitations

Despite significant advancements in neuroinflammation-on-a-chip technology there are several technical challenges and limitations that warrant further research. The development of an efficient organ-on-a-chip model presents complex challenges. The task requiring continuous collaboration between biologists and engineers as well as a high manufacturing cost is a bottleneck for widespread applications of these models^43^.

Additionally, due to the complexity of the central nervous system brain-on-a-chip models consist of different cell types that are often obtained from different donors. This is a less favoured situation^10^.

Even though multiple brain-on-a-chip models simulated successful neuroinflammatory changes, the key effector cells; peripheral immune cells often neglected pose another technical challenge. As a result, many studies are incomplete^10^.

Working with Induced pluripotent cells (iPS cells) poses limitations. These cells cannot usually exhibit terminal phenotypic differentiation, requiring additional simulations for validation^34^. Furthermore, IPS cells’ exact cellular identity are being questioned as a recent study found induced brain microvascular endothelial cells for a study of BBB found lacking functional attributes of typical endothelial cells^44^.

The slow adoption of new technologies is another limitation. Gathering substantial data is essential to convince the scientific community to transition from animal models to organ-on-a-chips models, requiring further research to optimize the design and validate its use in clinical studies^45^.

Lastly, cell sourcing is a challenge with options including commercial cell lines, primary tissues from donors, and iPSC sources. Commercial cell lines are convenient but may be subject to genetic or epigenetic drift over time^46^. Primary tissues are useful for rare disease studies but can be scarce, and obtaining healthy tissues ethically and practically is difficult^47^.

### Ethical considerations

As animal testing becomes ethically questionable, the organ-on-a-chip model offers an alternative but raises their own ethical dilemmas^45^.

Designed brain-on-a-chip models often prompt questions on how to categorize them and the protection they deserve^48^. A recent study discovered that in vitro neurons exhibited some kind of consciousness and sentience^49^. Without ethical standards governing this research area, however, we are left with the moral dilemmas where these models fall under the protection of. Human subjects or laboratory animals^48,50^.

The principle of autonomy, particularly obtaining informed consent is going to be a difficult task. Patients may need to consent without complete information as scientists are still exploring the true extent of risk and benefit of organ-on-a-chip model experimentation^48^. Researchers must follow strict regulations and ethical guidelines when conducting further research.

### Potential for scaling up and commercialization

Commercialization and scaling organ-on-a-chip models face various challenges. Standardization, reliability, cost-effectiveness, and regulatory compliance are essential to attract industrial interest^51^.

Despite limited initial capital investments in the field, the global organ-on-a-chip market is projected to grow, reaching $697.7 million by 2028. Companies like Emulate Inc., TissUse GmbH, MIMETAS, and CN Bio are actively advancing organ-on-a-chip models^43^.

Current manufacturing relies on soft lithography, which raises questions about reproducibility and scalability. A standardized, low-cost manufacturing method is imperative^51^.

3D bioprinting yields promising results, enabling the automated production of complex tissue structures with high fidelity, cost-effectiveness, and reproducibility. This one step will accelerate scaling and commercialization^43,51,52^.

### Future innovations and advancements in the field

The future holds exciting possibilities, technological advancements including multi-organs-on-a-chip or human-on-a-chip systems incorporate multiple organs and tissues, interconnected by channels, enabling in-depth analysis of disease processes, such as neuroinflammation in multiple sclerosis, in a dynamic context. Although in the early stages, multiple organ-on-a-chip models incorporating as many as ten organs have been constructed^53^. Advanced techniques such as 3D bioprinting to create more complex tissue architecture as well as real-time imaging integrated into the platform will allow a detailed understanding of many disease processes^53^.

Additionally, the integration of AI and machine learning with such systems will enable the analysis of vast, labour-intensive data sets, enhancing performance and revolutionizing healthcare developments^53^.

The potential avenues for further research in organ-on-a-chip technology include exploring alternative materials, reducing manufacturing costs, improving system components, developing better sensors, creating universal cell culture mediums, and addressing the challenges posed by the increasing complexity of multi-organ chips^53^.

Lastly, as we experiment and dissect the complex mechanisms underlying neuroinflammation, its progression, and potential avenue of therapeutic intervention using organ-on-a-chip technology, new clinical applications and intervention strategies will allow for an effective precision medicine, tailoring treatment to individual patients and effecting better disease outcome for many^10^.

## Conclusion

Damages of the protective myeline covering of the nerve fibres can occur due to MS. Understanding neuroinflammation processes remains essential to establish new MS treatments approaches. Various neurodegenerative diseases have common features. While the understanding of the brain response to pathogens arises, the specific triggers for the activation of glial cells and astrocytes remain unclear. This is where the significance of neuroinflammation becomes apparent, as it has eased the understanding and advancements of MS research. With newly identified AI and machine learning integration, neuroinflammation on a chip has proven its ability to detect specific changes in cell-to-cell communication that contribute to the development and progression of neuroinflammation.

This innovative approach has the potential to serve as a powerful tool for studying and screening brain inflammation, thus facilitating research into numerous neurodegenerative conditions.

By investigating the link between inflammation and a wide range of potential drug candidates, this platform aims to uncover genuine treatments. The utilization of brain chips promises to advance our understanding of the molecular processes involving BBB transporters, both in normal and diseased states. Further research needs to be addressed in order to enhance the performance and understanding of the neuroinflammation on a chip, hence aiming to provide more effective treatments for all CNS diseases.

## Ethical approval

Not applicable.

## Consent

Not applicable.

## Sources of funding

Not applicable.

## Author contribution

Conceptualization of ideas: all authors. Critical reviews with comments: all authors. Final draft: all authors approved the final manuscript.

## Conflicts of interest disclosure

Not applicable.

## Research registration unique identifying number (UIN)

Not applicable.

## Guarantor

Abubakar Nazir.

## Data availability statement

Not applicable.

## Provenance and peer review

Not commissioned, externally peer-reviewed.
